# The effects of a new device on mechanical complications of short peripheral intravenous catheters: A randomized controlled trial

**DOI:** 10.1177/11297298251389658

**Published:** 2025-11-11

**Authors:** Christopher Blacker, Ali-Reza Modiri, Gustaf Ljungman, Peter Frykholm

**Affiliations:** 1 Department of Women’s and Children’s Health, Uppsala University, Uppsala, Sweden; 2 Department of Surgical Sciences, Section of Anaesthesia and Intensive Care Medicine, Uppsala University, Uppsala, Sweden

**Keywords:** Catheterization, peripheral, mechanical complications, force-activated separation device, catheter dislodgement, randomized controlled trial, inventions, medical device

## Abstract

**Background:**

Placement of a short peripheral catheter (SPC) is one of the most common invasive procedures in healthcare. Catheter dysfunction occurs in up to 69%, caused by phlebitis, subcutaneous infiltration, occlusion, and dislodgement, conjointly considered as mechanical complications. Recent research has indicated that a new type of medical device, the force-activated separation device (FASD), may reduce these mechanical complications by 46%, but efficacy and safety data are lacking. The objective of this study was to investigate the efficacy and rate of adverse events (AEs) using a novel FASD.

**Method:**

This was a single center randomized controlled trial comparing a FASD to standard of care, where primary outcome was rate of mechanical complications for each arm. Adult patients, admitted to either a stroke ward or orthopedic ward at a University Hospital were eligible.

**Results:**

The population was mainly male, elderly, and had multiple co-morbidities. The study included 146 patients, contributing with a total of 194 catheters for the per protocol (PP) and 203 for the intention to treat (ITT) analyses. In the PP analysis, the total mechanical complication rate was 42% versus 46% (*p* = 0.56), while the dislodgement rate was 3.4% versus 12% (*p* = 0.04) in the intervention and control group, respectively. For ITT, total mechanical complication rate was 42% versus 47% (*p* = 0.47) and the dislodgement rate was 4.2% versus 13% (*p* = 0.03) in the intervention and control groups. FASD implementation resulted in a 73% reduction in dislodgements in the PP, and a 68% reduction in the ITT analysis. No device related AEs were recorded.

**Conclusion:**

The implementation of a novel FASD did not demonstrate a reduction of total mechanical complications, however, a 73% reduction in dislodgements in the PP, and a 68% reduction in the ITT analysis was noted. No device-related AEs were recorded.

Clinical Trials; clinicaltrials.gov; ID: NCT05814887.

## Introduction

**Figure 1.**
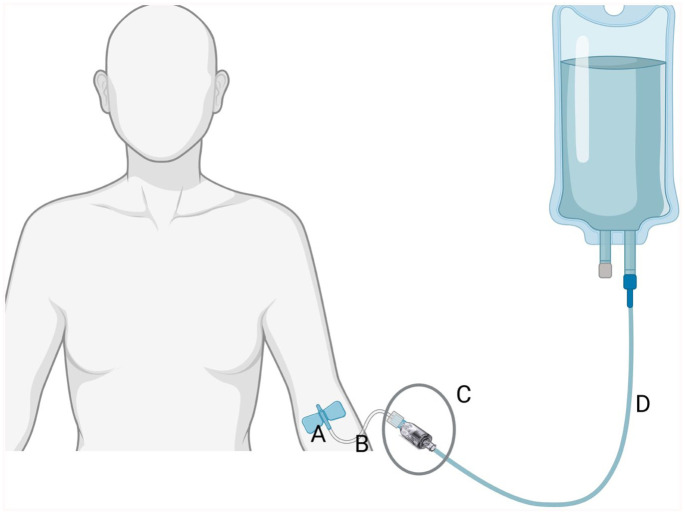
Schematic drawing of placement of investigational device during the trial. A = SPC, B = 10 cm short extension line, C = Investigational device in connected state, D = 175 cm Infusion set. Gray circle indicates investigational device. SPC: Short Peripheral Catheter. According to manufacturer instructions for use the investigational device shall be used together with an extension line between 5 and 25 cm in length.

An estimated 1–2 billion short peripheral catheters (SPC) are used world-wide yearly.^1^ Among hospitalized patients, up to 90% receive a SPC; insertion is one of the most common invasive procedures in hospitalized patients.^2^ Reports of catheter dysfunction rates range from 36% to 69%.^3–9^ The causes are attributed to phlebitis in 0.7—63%, subcutaneous infiltration in 16—34%, occlusion in 8—22%, and dislodgement in 4—18%.^2–4,7,9–14^ Estimates of costs of up to seven billion USD in the USA for these types of failures have been reported.^15^ Previous studies have suggested that mechanical irritation, that is, repeated pulling and pushing on the catheter could be an important factor resulting in SPC failure.^16–18^ Therefore, there is a strong incentive to develop new methods for prevention of mechanical complications, where one strategy could be to reduce mechanical forces acting on the SPC. Recent research suggests that a Force-Activated Separation Device (FASD) could potentially reduce SPC failures up to 46%.^15^ In clinical settings, the FASD device is connected between the patient’s intravenous (IV) infusion set and an extension line (which in turn is connected to the SPC, Figure 1). It will separate when a force above a threshold is applied to the infusion set, thereby reducing the mechanical forces acting on the SPC.

FASD is a new technology, with little published data about safety and efficacy. Therefore, the aim of this study was to investigate the safety and efficacy of a new FASD in reducing the number of mechanical complications during IV therapy in an adult hospitalized population.

## Materials and methods

This was a single center randomized open controlled trial conducted at the University Hospital of Uppsala, Sweden. The study was approved by the Swedish Ethical Review Authority (2022-06847-01) and the Swedish Medical Products Agency (CIV-23-03-042558). The trial was registered at clinicaltrials.gov (NCT05814887) April 14th 2023. The trial adhered to the CONSORT statement.^19^

### Eligibility criteria

Patients eligible for the trial were ⩾18 years of age, having a SPC in place for intravenous (IV) infusion therapy, fluent in the Swedish language, SPC without signs of mechanical complication and a signed informed consent. Exclusion criteria were SPC to be used for sampling only, pregnancy, inability to attain informed consent, patients under palliative care, or SPC for only bolus IV infusions. Bolus IV infusions were defined as an infusion time of <15 min.

The study was conducted at two orthopedic wards and one stroke ward.

### Outcomes

The primary outcome was to investigate the rate of mechanical complications of an IV therapy session using a FASD (ReLink^®^) compared to using current standard of care (SOC). Mechanical complications were in the study defined as phlebitis, subcutaneous infiltration, occlusion, and dislodgement. SOC was defined as using aseptic technique, choice of optimal SPC size based on vessel and planned usage (favoring smaller SPC diameter), choice of optimal placement site (favoring lower arm), securing with high permeable polyurethane dressings, documentation of the procedure, SPC inspection every 8 h for complications and dressing status, and daily revision of clinical indication leading to removal if no clinical indication or after 72 h.^20–23^ Possible adjuncts include infrared vein scanners.

Secondary outcomes were to investigate the frequency and severity of any adverse events (AE), healthcare workers opinion of the new FASD, and the cost of SPC replacement for the hospital in terms of man-hours and other resources.

AE was defined as any untoward medical occurrence, unintended disease or injury or any untoward clinical signs (including abnormal laboratory findings) in subjects, users or other persons whether or not related to the investigational medical device.

### Intervention: ReLink^®^ device

The ReLink^®^ Care (Interlinked AB) is a FASD designed to be connected to a SPC to reduce the risk of mechanical complication during IV therapy (Figure 1). It acts as a weak link between the SPC (with an extension line) and the infusion set. If sufficient force is applied to the infusion set, the ReLink^®^ device will separate into two parts, thereby preventing dislodgement of the SPC. The device contains dual valves, which automatically close when disconnected, thereby preventing flow of blood and fluids.

### Randomization

Randomization was done in a ratio of 1:1 in blocks of four. Patients were randomly assigned to belong to either the intervention group or control group through computerized randomization. Pre-printed, concealed, numbered randomization envelopes indicating intervention or control were prepared and distributed to the wards. Randomization sequence was generated by the first author (CB) and stored in a secure location. Implementation was completed by either nurses at the wards, CB, or the research coordinator (ARM). Patients could be included multiple times in case of planned or inadvertent removal of the SPC. Blinding was not implemented.

In patients randomized to the intervention group, a ReLink^®^ device was added to the infusion set, while in the control group, no ReLink^®^ was attached. Once a separation of the investigational device in the interventional arm occurred, a new investigational device was used.

### SPCs and infusion sets

All SPCs were placed by nurses at either the emergency department, ambulance services, or the investigating wards. The procured SPCs were BD Venflon™ Pro Safety (several sizes) fixed by a 3M Tegaderm™ I.V advanced securement dressing. The infusion set was a 175 cm CODAN green line^®^ L86-R, with, in most cases, a BD Connecta™ 10 cm extension line, supplemented in varying degrees with a BD MaxZero™ needle-free connector. Data on placement was found in the electronic medical records and/or directly reported by the nursing staff.

All patients were monitored daily for the occurrence of mechanical complications with responsible staff and electronic medical records, until planned termination or a mechanical complication occurred.

### Healthcare worker questionnaire

A questionnaire was created to obtain information about healthcare workers’ opinion of the device. Information collected from the staff was gender, age, profession, and years of service. The respondents were asked to rate (1) willingness to use the device again; (2) willingness to recommend the device to a colleague; (3) willingness for relatives to have access to the device in case of hospitalization. Each statement was graded from zero (strongly disagree) to five (strongly agree). The questionnaire (Supplemental Figure 1. Questionnaire) was made available through printed copies at the wards and in the employee newsletter.

### Adverse events

AEs were followed non-systematically and daily by inspecting the SPC, asking the attending nurse and reviewing electronic medical records. An AE form was freely available in the ward and oral and written instructions for use were provided. The AE form is available in the supplementary information (Supplemental Table 1. Adverse events).

### Cost analysis

Only direct costs for the hospital were included in this study. All costs are in Euro (€) and from 2023 pricing lists (based on SEK to € conversion rate from December 27th 2023).^24^

Costs were calculated based on material usage and staff costs. Material costs per unit were obtained from Uppsala University Hospital Procurement database (Agresso). Hospital staff were asked to fill out the time required to place a new IV access in minutes once a mechanical complication occurred and a new IV access was required.

### Statistical methods

The sample size was calculated with an alpha error of 0.05 and a power of 80%, with a 30% potential loss of data, resulting in a final sample size of 200 patients.

Student’s two-sided *t*-test was used for continuous variables (age, time), Chi-square tests for the dichotomous variables, unless ⩽5 cases, in which case Fisher’s exact test was used. A *p*-value of <0.05 was considered significant.

To address the methodological problem of multiple SPCs for some of the patients, a sensitivity analysis was performed using only the first inserted SPC.

The analysis was done using SPSS version 28 statistical analysis package (IBM, Armonk, NY, USA).

A post-hoc SPC survival analysis using Kaplan-Meier diagrams was conducted analyzing SPC dwell time. Dwell time was defined as the duration of safe function of the SPC. Log Rank test was used to determine differences.

## Results

### Recruitment

**Figure 2.**
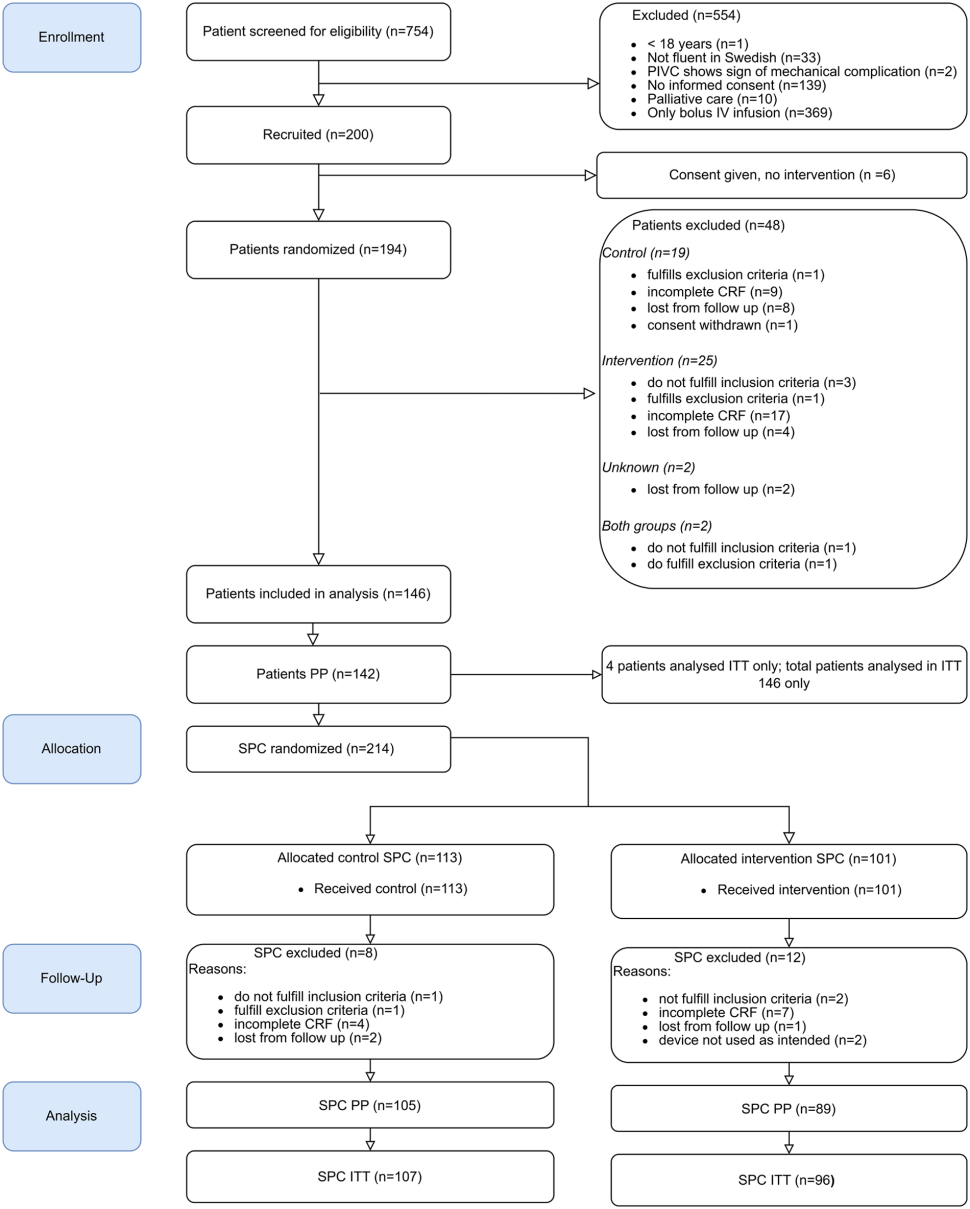
CONSORT flow chart. CRF: case report form; ITT: intention to treat; PP: per protocol, SPC: short peripheral catheter.

From May 4th 2023 until November 12th 2023, 754 patients were screened for eligibility, of which 200 patients were enrolled in the trial. Reasons for exclusion are displayed in Figure 2.

Of the 200 patients enrolled in the trial, an additional 54 were excluded (Figure 2). Data from a total of 146 patients was available, of which 4 had per protocol (PP) infringements and were included only in the intention to treat (ITT) analysis together with data from PP. From PP patients, data from a total of 214 SPCs was collected, of which 20 were excluded. For the primary analysis, a total 194 SPC were available, 89 in the intervention and 105 in the control group. For the ITT analysis a total of 203 SPC were available, 96 in the intervention and 107 in the control group.

### Patient characteristics

Patient characteristics are summarized in Table 1. There were no significant differences between the groups, except for more cases of cancer in the intervention group (*p* = 0.04). For admission details see supplementary information (Supplemental Table 2. Admission cause).

**Table 1.** Clinical characteristics of the study population.

		Control		Intervention		*p* Value
Age (years)		68 (15)^^^	26–92^^^	69 (14)^^^	19–91^^^	0.90
Gender	Male	63	61%	51	57%	0.58
Cardiovascular disease	Yes	47	45%	37	41%	0.57
Stroke	Yes	14	14%	12	13%	0.98
Diabetes	Yes	33	32%	27	30%	0.80
Hypertension	Yes	65	63%	63	70%	0.27
Cancer	Yes	17*	16%	26*	29%	0.04
COPD	Yes	7 _b_	6.7%	5 _b_	5.6%	0.78
Other pulmonary disease	Yes	20	19%	21	23%	0.49
Kidney disease	Yes	19	18%	22	24%	0.29
Liver disease	Yes	9 _b_	8.7%	3 _b_	3.3%	0.15
Neurologic disease	Yes	16	15%	14	16%	0.97
Chronic pain	Yes	9 _b_	8.7%	5 _b_	5.6%	0.58
Obesitas	Yes	28	27%	31	34%	0.26
Malnourished	Yes	7 _b_	6.7%	3 _b_	3.3%	0.34
Other	Yes	27	26%	28	31%	0.43
Number of chronic diseases	None	13	13%	6	6.7%	0.45
	1–2	36	35%	31	35%	
	3	28	27%	25	28%	
	4–6	23	22%	20	22%	
	⩽7	4	3.8%	8	8.9%	
Smoker	Yes	9 _b_	8.7%	2 _b_	2.2%	0.07
ADL dependency	Yes	15	14%	12	13%	0.83
Ward	Orthopedic ward	90	87%	77	86%	0.84
	Stroke ward	14	14%	13	14%	
Admission	Emergency	98	94%	86	96%	0.75
	Elective	6 _b_	6%	4 _b_	4%	
Admissions cause	Neurological disease	14	14%	11	12%	0.44
	Infection	24	23%	29	32%	
	Orthopedic	58	56%	41	47%	
	General surgical	8	7.7%	8	8.9%	
	Other	0	0%	1	1.1%	
Total patients	*n*	104		90		

BMI: body mass index; ADL: activities of daily living; *n*: number of patients; %: subdivision in percent for respective category.

Data are presented as mean (standard deviation), followed by range, or numbers of patients followed by percentages. Subscript b: Fisher’s exact test.

^ Continuous variable. Student’s two-sided *t*-test was used for continuous variables, Chi-square tests for the remaining dichotomous variables.

* *p* < 0.05.

### SPC characteristics

The average SPC duration was 96 (86) h for the control group, and 80 (66) for the intervention group (*p* = 0.17). Clinical characteristics are displayed in Table 2.

**Table 2.** SPC characteristics.

		Control		Intervention		*p* Value
SPC duration (h)		96 (86)^^^	3–422	80 (66)^^^	2–310	0.17
SPC type	24G	1	1.0%	0	0.0%	0.98 _b_
	22G	20	19%	18	21%	
	20G	40	39%	35	40%	
	18G	39	38%	33	38%	
	17G	4	3.8%	2	2.3%	
	16G	0	0.0%	0	0.0%	
	14G	0	0.0%	0	0.0%	
SPC placement	Back of hand	19	18%	25	28%	0.43 _b_
	Inner wrist	5	4.5%	5	5.6%	
	Forearm	43	41%	26	29%	
	Antecubital fossa	32	30.5%	29	32.6%	
	Upper arm	1	1.0%	1	1.1%	
	Leg	2	1.9%	0	0.0%	
	Foot	3	2.9%	3	3.4%	
	Other	0	0.0%	0	0.0%	
Side	Left	56	53.8%	48	46.2%	0.93
	Right	49	53.3%	41	53.9%	
	Total	105	46.7%	89	46.1%	

SPC: short peripheral catheter; G: gauge; %: subdivision in percent control and intervention per row.

Data are presented as mean (standard deviation), followed by range, or number of SPC followed by percentages. Student’s two-sided *t*-test was used for continuous variables, Chi-square tests for the remaining dichotomous variables. Subscript b: Fisher’s exact test.

^ Continuous variable.

### Primary outcome

For the PP analysis, 194 SPCs were included, in which 85 complications occurred. A statistically significant difference was found for dislodgement (3.4% vs 12.4%) favoring intervention (*p* = 0.04), the remaining complications were non-significant (Table 3).

**Table 3.** Complications.

	Per protocol					Intention to treat				
	Control		Intervention			Control		Intervention		
	Count	%	Count	%	*p*	Count	%	Count	%	*p*
Planned termination	57	54	52	58	0.56	57	53	56	58	0.47
Infiltration	5	4.8	10	11	0.11 _b_	5	4.7	10	10	0.19 _a_
Phlebitis	4	3.8	4	4.8	1.0 _b_	4	3.7	4	4.2	1.0 _a_
Dislodgement	13	12	3	3.4	0.04 _a_*	14	13	4	4.2	0.03 _a_*
Occlusion	12	11	5	5.6	0.20 _a_	12	11	5	5.2	0.07 _a_
Leakage	8	7.6	11	12	0.27	9	8.4	12	13	0.34
Pain at injection site	6	5.7	4	4.5	0.76 _a_	6	5.6	5	5.2	1.0 _b_
Total complications	48	46	37	42	0.56	50	47	40	42	0.47

Subscript a: Fisher’s exact test, remaining Chi-Square.

* *p* < 0.05.

For dislodgements, the absolute risk reduction and relative risk reduction were 9.0% and 73%, respectively, favoring intervention in the PP analysis. The number needed to treat to avoid one dislodgement was 11, see supplementary information (Supplemental Table 3. NNT).

### ITT

An ITT analysis was performed. Data from 203 SPCs were available, of which 90 complications occurred (Table 3). Significant results were found for dislodgement (4.2% vs 13.1%) favoring intervention (*p* = 0.03), the remaining complications were non-significant (Table 3).

For dislodgements, absolute risk reduction and relative risk reduction was 8.9% and 68%, respectively, favoring intervention in the ITT analysis. The number needed to treat to avoid one dislodgement was 11 (Supplemental Table 3. NNT).

### Sensitivity analysis

In the sensitivity analysis only the first inserted SPC for the 143 patients (descriptive statistics available in Supplemental Table 4) were included. Dislodgement was less common in the intervention group (3.2% vs 12% in controls), *p* = 0.046 (Supplemental Table 5).

### Post-hoc analysis

A Kaplan-Meier analysis of mechanical complications data for the PP population was performed. No statistical significance was found between the groups with regards to SPC dwell time (*p* = 0.81). Neither were significant differences found for each of the individual mechanical complications using subgroup Kaplan-Meier analysis (Supplemental Figures 2–9).

### Adverse events

No AEs related to the device were recorded.

### Healthcare staff questionnaire

A total of 19 (44.2%) respondents answered the questionnaire. Respondents were 73.7% female, mean age 39.1 years, and all nurses; 42.1% had less than 5 years of experience, 5.3% 5–10 years, 10.5% 11–20 years, and 10.5% more than 20 years. Average results for question 1, 2, and 3 were 3.6, 4.0, and 4.0 respectively (Supplemental Table 6).

### Costs

Cost data was available for 45 mechanical complications with an average cost in materials of €2.11, subdivision of articles in Supplemental Table 7.

**Figure 3.**
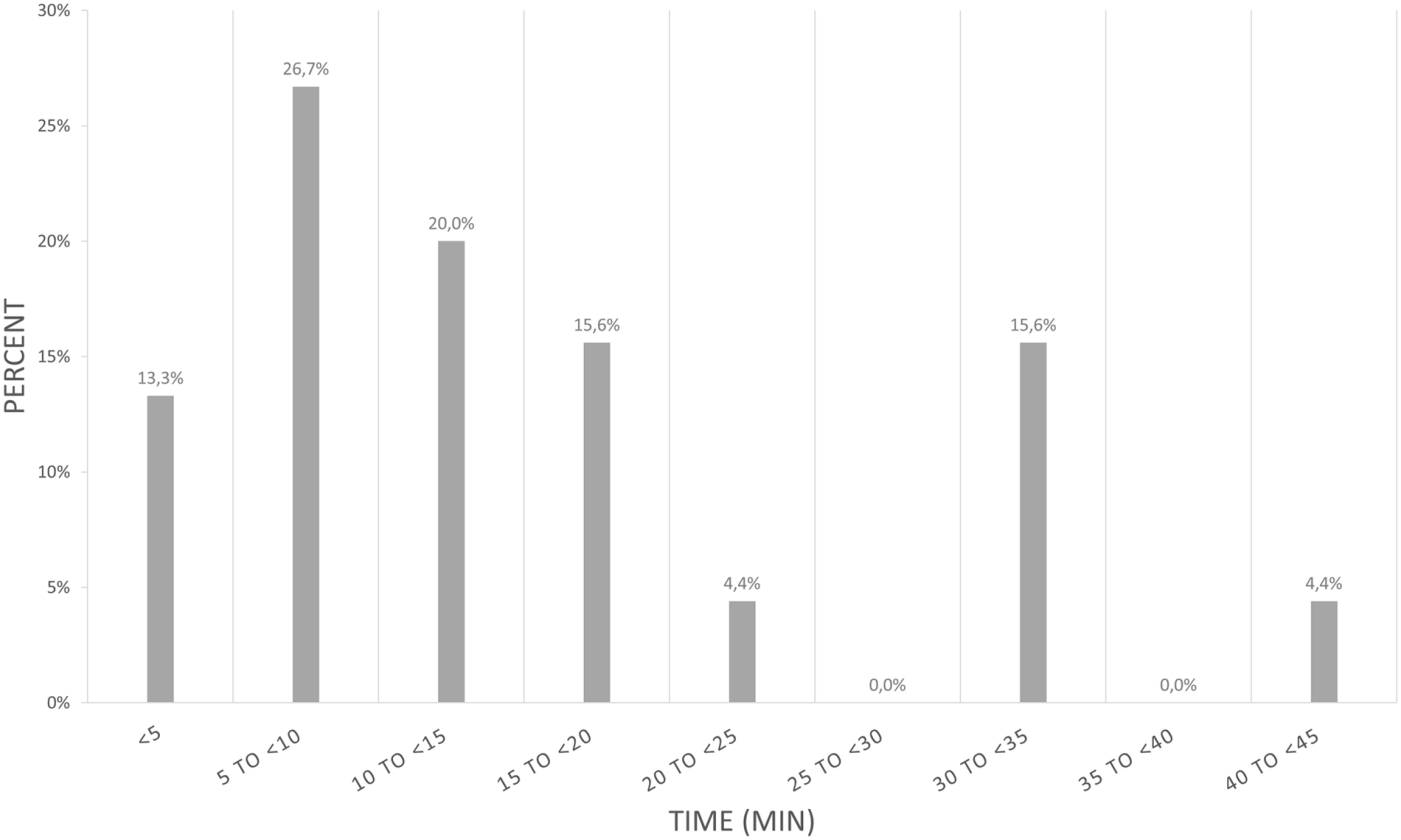
Distribution of time (min) to reestablish IV access.

Time spent to place a new SPC was on average 13 min, with distributions demonstrated in Figure 3.

## Discussion

The rate of mechanical complications was compared for SPCs with or without the ReLink© FASD. Although there was no statistically significant difference in the total complication rate, a significant reduction of dislodgements was found in the intervention group; 73% (absolute risk reduction 9.0%) in the PP and 68% (absolute risk reduction 8.9%) in the ITT analysis. To the best of the authors’ knowledge, this is the first clinical trial to demonstrate a reduction in dislodgements using a FASD.

Previous studies on FASDs are scarce. Panza et al. reported a 46% reduction of a composite measure of all mechanical complications using another FASD.^15^ However, they did not demonstrate a significant difference in dislodgement reduction. Reasons for these differences could lie in the SOC procedures at the different sites. Panza et al. used the PIV5Rights© as a SOC. The PIV5Rights©, constitutes the usage of vascular access teams and optimized SPC insertion and management procedures, including: skilled inserters with ⩾90% first pass success and competent in ultrasound SPC access in order to avoiding “blind sticks,” choosing vessel in forearm and avoiding joint near vessels, optimized vein-catheter ratio, routine assessment for clinically indicated SPC placement, and routine pulsatile flushing and dressing changes ever seventh day.^25^ Usage of different SOC could reflect the differences in the total complication rates, where using the PIV5Rights© could play an important role compared to Swedish SOC. The differences in SOC could explain the low number of dislodgement events in the Panza et al. group, resulting in a too small sample size to detect any significant differences in the dislodgment rates.

Furthermore, securement of the extension line was used in the Panza et al. study. At the study site, the investigation device hung freely, potentially adding a small weight to the infusion system and more strain on the SPC, resulting in increased risk of catheter tip movement. This could possibly explain the non-significant higher incidence of infiltration and leakage in the intervention arm. The association between catheter tip movement inside the vessel and increased risk of edema has been previously studied, and is proposed as a likely cause of infiltration by the Infusion Nurses Society.^13,27^ Further reasons for different results between this study and Panza et al. could lie in the study population, who were older than those in Panza et al. (68 ± SD 16 vs 60.1 ± SD 15.9 respectively), though unlikely as age does not seem to be an independent risk factor.^6,26^

A FASD is a weak link in the infusion system that will separate at a predetermined force. Published data state this force to be 18 ± 4.5 N.^15^ Thus, the likelihood of a FASD separating under this threshold is small. Therefore, as long as the FASD is in its connected state, the forces exerted on the SPC should theoretically be similar to those of a SPC without FASD. Phlebitis, infiltration, and leakage have been postulated to result from micromovements within the vessel.^17,27–29^ These micromovements may not be reduced by using a FASD. In contrast, dislodgements of the SPC or macro movements, require a stronger force, which thus suggests the main beneficial effect of a FASD.

Furthermore, healthcare workers’ opinions of the investigational device were analyzed, receiving an average score of 3.6 out of 5 that they would use the device again, indicating that there was an acceptance for the FASD technology. This was even more prominent when asked if they would recommend the device to colleagues (4 out of 5) and if they would like to have their relatives use a FASD (4 out of 5), suggesting that nurses appreciate the advantage of FASDs. However, due to the low response rate (44.2%) and possible placebo effects, these results should be interpreted with caution.

There were no AEs reported related to the device. Similarly, Panza et al. found no severe AEs related to the use of their studied device.^15^ This study strengthens the evidence that the use of FASDs are likely to be safe.

With regards to cost data, only 50% (45 out of 90) of the mechanical complication events were available for analysis. The average time to replace a SPC was reported to be 13 (10) min. However, this was self-reported by the practicing nurse and should probably be viewed as the lower limit of time spent on SPC reinsertion. Forty percent of SPCs were replaced in less than 10 min. However, the time for nursing staff to detect and replace a non-functional SPC was more than 20 min in 24% of the cases. These results are consistent with a previous report in which 39% of SPC replacements required ⩽10 min, yet exceeded 20 min in 22% of cases.^30^

The average material cost for SPC placement was €2.11. In contrast, material cost for SPC replacement in the USA has been reported to be USD5.51. The difference could be explained by the procurement system in Swedish resulting in lower pricing in Europe compared to the USA.^31^ Patients with difficult SPC placement have been estimated to cost USD84 per SPC replacement in material and staff resources.^31^ The results from this study reinforce that SPC placement is a difficult and time-consuming procedure in up to one in five cases. Thereby, FASDs could potentially reduce both material and personnel cost, especially for individuals with risk of dislodgements, though this would need to be further investigated.

Finally, the post-hoc Kaplan-Meier analysis indicated no difference in the total SPC dwell time between treatments with or without the device (*p* = 0.57).

A strength of this study lies in the randomized design, where the only intervention was implementation of the FASD. The study was conducted in a tertiary hospital providing both standard and specialized care. The study population was diverse in both age and gender, most patients had multiple comorbidities (88% ⩾1 comorbidity; 56% ⩾3 comorbidities), making this study generalizable to a typical adult healthcare setting. The results hold up in a sensitivity analysis where only the first SPC per patient was included.

Limitations of the study lie in the loss of patient and SPC data reflected both in the PP and ITT. Multiple SPCs per patient might influence Type 1 errors, though this was addressed by performing a sensitivity analysis. Furthermore, blinding was not an option due to the nature of the investigation.

Since the company producing the FASD has not yet set a price, it was not possible to do a cost-benefit analysis.

## Conclusion

In conclusion, the novel FASD may reduce the rate of SPC dislodgement, although no reduction in the total rate of SPC mechanical complications could be demonstrated. The device was considered safe to use as no device-related AEs were recorded.

## Supplemental Material

sj-pdf-1-jva-10.1177_11297298251389658 – Supplemental material for The effects of a new device on mechanical complications of short peripheral intravenous catheters: A randomized controlled trial Supplemental material, sj-pdf-1-jva-10.1177_11297298251389658 for The effects of a new device on mechanical complications of short peripheral intravenous catheters: A randomized controlled trial by Christopher Blacker, Ali-Reza Modiri, Gustaf Ljungman and Peter Frykholm in The Journal of Vascular Access
