# Supplementary material for: The effects of a new device on mechanical complications of short peripheral intravenous catheters: A randomized controlled trial
Source: J Vasc Access. 2025 Nov 11;27(3):1179–88. doi: 10.1177/11297298251389658 (PMC13135644; doi:10.1177/11297298251389658)
Supplement: sj-pdf-1-jva-10.1177_11297298251389658 – Supplemental material for The effects of a new device on mechanical complications of short peripheral intravenous catheters: A randomized controlled trial [file sj-pdf-1-jva-10.1177_11297298251389658.pdf]

Supplemental information:

S. Fig 1. Questionnaire

ReLink® - User Questionnaire

Thank you for participating in the study "Reduction of mechanical complications using a new medical technology product." As a complement to your participation, we would like to have an idea of what you think about using ReLink®. Your answers will be treated completely anonymously.

Please fill in the following information by circling the response that best fits you.

Gender: Male Female Prefer not to disclose

Your age: ____________

Your profession: Nurse Nurse assistant Doctor Other: _______________

Years in the profession: <5 5 – 10 11 - 20 >20

Describe how well the following statements agree or disagree with your experience of the product ReLink® (circle your answer):

I would use the product again

Strongly disagree (0) 1 2 3 4 (5) Strongly agree

I would recommend the product to a colleague

Strongly disagree (0) 1 2 3 4 (5) Strongly agree

I would want my relatives to have access to the product in case of potential healthcare needs

Strongly disagree (0) 1 2 3 4 (5) Strongly agree

Comments:

____
____
____
____

Thank you for your participation!

S. Table 1. Adverse events

Reduction of Mechanical Complications through the Use of a New Medical Device

This adverse event form aims to gather information about all adverse events from all participants in the clinical study related to the use of ReLink®.

Name:____

Social Security Number: ______________________Department:__________________________

Severity Level	Related to ReLink®	Actions Taken	Outcome of Adverse Event	Expected Adverse Event	Severe Adverse Event?
**1 = Mild**	1 = Unlikely	0 = None	0 = None	1 = Yes	1 = Yes
**2 = Moderate**	2 = Possibly related 3 = Probably related	1 = Medical intervention	1 = Medical intervention	2 = No	2 = No
**3 = Severe**			3 = Prolonged hospital stay		
**4 = Patient's life and health affected**	4 = Related	3 = Prolonged hospital stay	4 = Discontinued study participation		
		4 = Discontinued study participation	5 = Other		
		5 = Other			

Adverse Event	Observation Date	Date of Resolution	Severity Level	Relation to ReLink®	Actions Taken	Outcome of Event	Expected Adverse Event	Severe Adverse Event

Thank you for your participation!

S. Table 2. Admission cause

		Count	%
Admission reason	Abdominal pain	1	0.7%
	Ankle fracture	6	4.1%
	Appendicitis	3	2.1%
	Backpain	2	1.4%
	Cellulitis	1	0.7%
	Cholecystitis	1	0.7%
	Confusion	1	0.7%
	Elbow luxation	1	0.7%
	Elbow surgery	1	0.7%
	Extremities amputation	7	4.8%
	Extremities laceration	1	0.7%
	Foot fracture	2	1.4%
	Gastric retention	1	0.7%
	Gastrointestinal bleeding	1	0.7%
	Hand fracture	1	0.7%
	Hand injury	1	0.7%
	Hip arthrosis	1	0.7%
	Hip luxation	1	0.7%
	Hip fracture	35	24.0%
	Icterus	1	0.7%
	Ileus	3	2.1%
	Infected diabetic foot	4	2.8%
	Infected hip prothesis	1	0.7%
	Infected prothesis	2	1.4%
	Intracranial hemorrhage	3	2.1%
	Knee fracture	1	0.7%
	Lower leg fracture	3	2.1%
	Necrotic skin lesion	1	0.7%
	Nystagmus	1	0.7%
	Odema	1	0.7%
	Pancreatitis	1	0.7%
	Pelvic fracture	1	0.7%
	Perineal abscess	1	0.7%
	Plantar abscess	2	1.4%
	Pneumonia	1	0,7%
	Post trauma arthrosis	1	0.7%
	Post-op bleeding	1	0.7%
	Post-op infection	7	4.8%
	Prothesis infection	5	3.5
	Quadriceps rupture	2	1.4%
	Revision hip replacement	1	0.7%
	Septic arthritis	7	4.8%

	Scoliosis surgery	1	0.7%
	Spinal stenosis	4	2.7%
	Spine fracture	4	2.7%
	Stroke	8	5.5%
	Talus necrosis	1	0.7%
	Tendovaginitis	3	2.1%
	Transient ischemic attack	1	0.7%
	Upper arm fracture	3	2.1%
	Vertigo	2	1.4%
	Volvulus	1	0.7%

S. Table 3. NNT

	Outcome Present	Outcome Absent	
Treated	a =	b =	EER (Experimental Event Rate) $\frac{a}{(a+b)} =$
Control	c =	d =	CER (Control Event Rate) $\frac{c}{c+d} =$

Relative Risk Reduction (RRR)	Absolute Risk Reduction (ARR)	Numbers Needed to Treat (NNT)
$\frac{CER - EER}{CER} = RRR$	$CER - EER = ARR$	$\frac{1}{ARR} = NNT$

Source: [1]

S. Table 4 – Descriptive statistics of sensitivity analysis population

		Control		Intervention		p value
Age (years)		69 (15)^	26 - 92^	67 (16) ^	19 - 91^	0.75
Gender	Male	48	59%	32	52%	0.36
Cardiovascular disease	Yes	34	42%	22	36%	0.43
Stroke	Yes	9	11%	10	16%	0.38
Diabetes	Yes	26	32%	15	24%	0.30
Hypertension	Yes	49	61%	42	68%	0.37
Cancer	Yes	12	15%	15	24%	0.16
COPD	Yes	5 $_b$	6.2%	4 $_b$	6.5%	1.0
Other pulmonary disease	Yes	13	16%	8	13%	0.60

Kidney disease	Yes	13	16%	11	18%	0.79
Liver disease	Yes	4 b	4.9%	2 b	3.2%	0.70
Neurologic disease	Yes	13	16%	10	16%	0.99
Chronic pain	Yes	8 b	9.9%	5 b	4.8%	0.35
Obesitas	Yes	21	26%	18	29%	0.68
Malnourished	Yes	4 b	4.9	2 b	3.2%	0.70
Other	Yes	22	27%	16	26%	0.86
Number of chronic diseases	None	11	14%	6	9.7%	0.87
	1 to 2	24	30%	23	37%	
	3	15	19%	11	18%	
	4 to 6	29	36%	20	32%	
	≤ 7	2	2.5%	2	3.2%	
Smoker	Yes	9 b	11%	2 b	3.2%	0.11
BMI		27 (4.9) ^	17 - 39^	28 (6.3) ^	17 - 45^	0.042*
ADL dependency	Yes	11	14%	8	13%	0.91
Ward	Orthopedic ward	72	89%	52	84%	0.38
	Stroke ward	9	11%	10	16%	
Admission	Emergency	76	94%	58	94%	1.0
	Elective	5 b	6.2%	4 b	6.5%	
Admissions cause	Neurological disease	9	11%	8	13%	0.78
	Infection	16	20%	14	23%	
	Orthopedic	48	59%	34	55%	
	General surgical	8	9.9%	5	8.1%	
	Other	0	0%	1	1.6%	
Total patients	n	81		62		

Data are presented as mean (standard deviation), followed by range, or numbers of patients followed by percentages. BMI, body mass index. ADL, activities of daily living. n = number of patients. % = subdivision in percent for respective category. ^ = continuous variable. Student´s two-sided t-test was used for continuous variables, Chi-square tests for the remaining dichotomous variables. Subscript $_b$ = Fisher's exact test. * = p<0.05.

S. Table 5. Sensitivity analysis

		Control		Intervention		
		Count	%	Count	%	p
	Complication	35	43%	21	34%	0.26
Type of Complication	Planned removal	46	57%	41	66%	
	Infiltration	4	4.9%	6	9.7%	
	Phlebitis	2	2.5%	2	3.2%	
	Dislodgement	10	12%	2	3.2%	0.046 $_b$ *
	Occlusion	9	11%	3	4.8%	
	Leakage	6	7.4%	6	9.7%	
	Pain at injection site	4	4.9%	2	3.2%	
	Total	81		62		

Descriptive statistics for the first PIVC from each patient. Percentage represent % distribution control and intervention per reason for PIVC removal. Subscript $_b$ = Fisher exact test, remaining Chi-Square. Significance * = p<0,05.

S. Fig 2. Mechanical complication

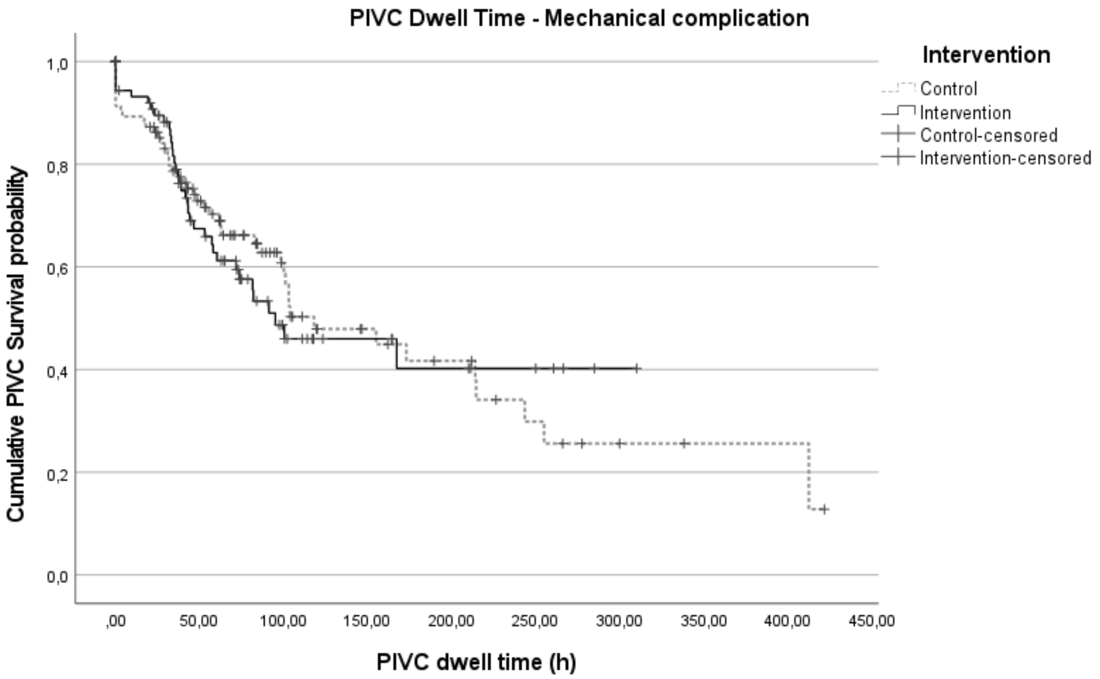

Fig 2. Kaplan-Meier diagram for all mechanical complications, where Y axis indicate probability of PIVC survival (1 = 100% survival, 0 = 0% survival). X axis is dwell time in hours (h). Grey dotted line represent control, black whole line represent intervention, crosses on the line (censoring) represent a PIVC was removed before the longest PIVC dwell time observation. Log Rank p=0.811

S. Fig 3. Planned termination

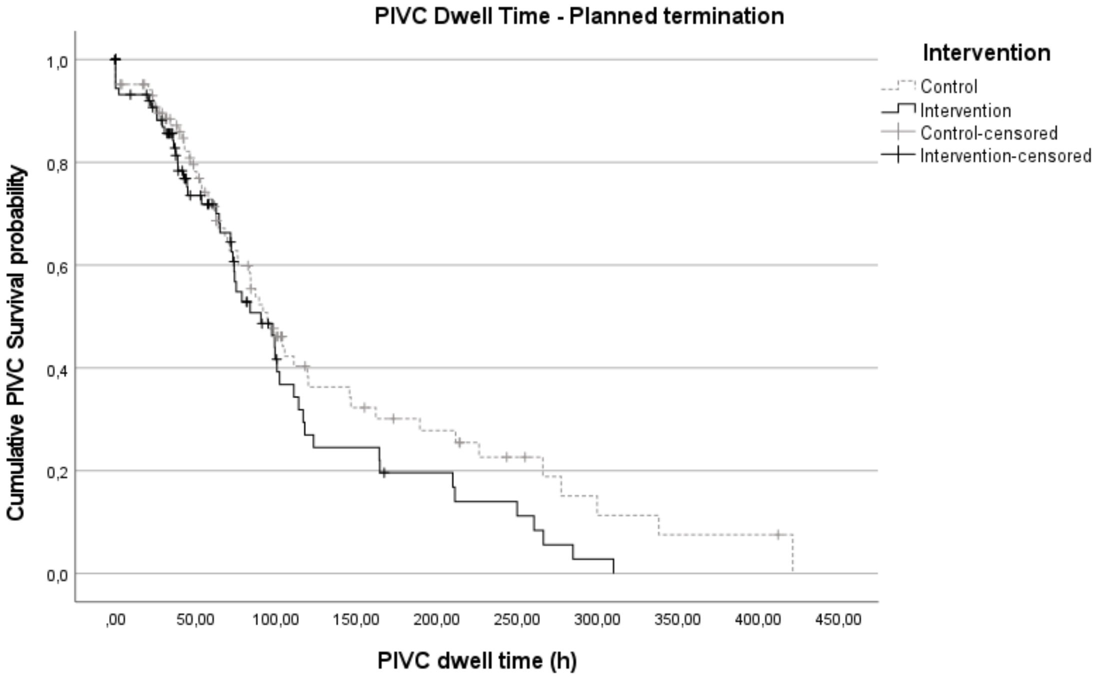

Fig 3. Kaplan-Meier diagram for all planned termination, where Y axis indicate probability of PIVC survival (1 = 100% survival, 0 = 0% survival). X axis is dwell time in hours (h). Grey dotted line represent control, black

whole line represent intervention, crosses on the line (censoring) represent a PIVC was removed before the longest PIVC dwell time observation. Log Rank test p=0.131.

S. Fig 4. Infiltration

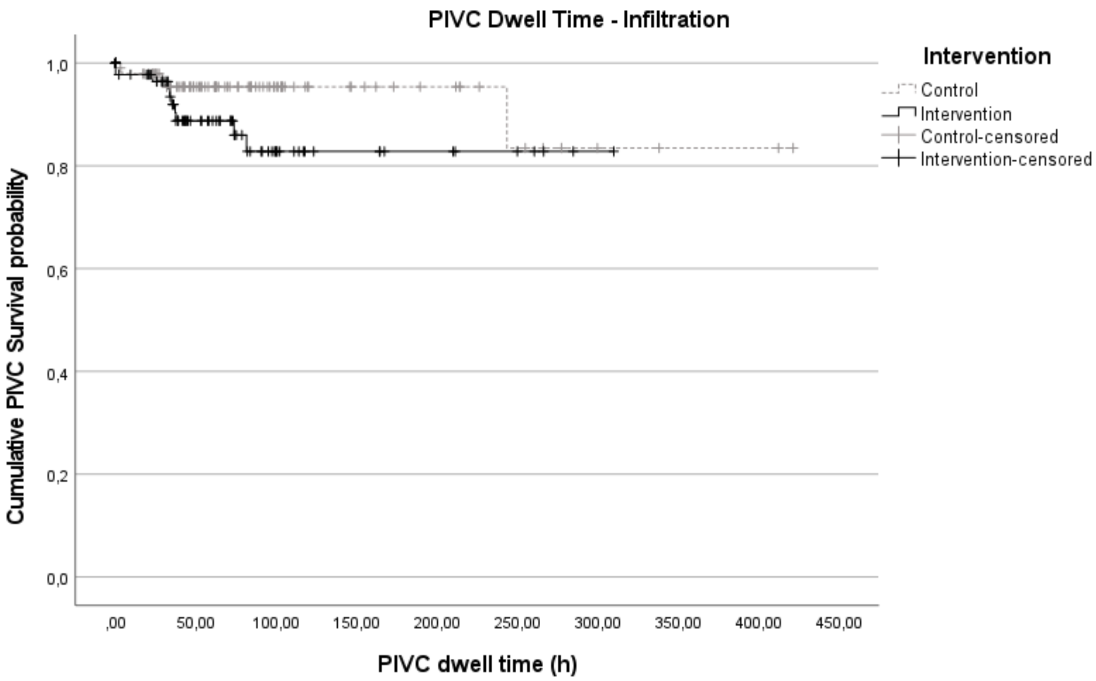

Fig 4. Kaplan-Meier diagram for all infiltration, where Y axis indicate probability of PIVC survival (1 = 100% survival, 0 = 0% survival). X axis is dwell time in hours (h). Grey dotted line represent control, black whole line represent intervention, crosses on the line (censoring) represent a PIVC was removed before the longest PIVC dwell time observation. Log Rank test p=0.092.

S. Fig 5. Phlebitis

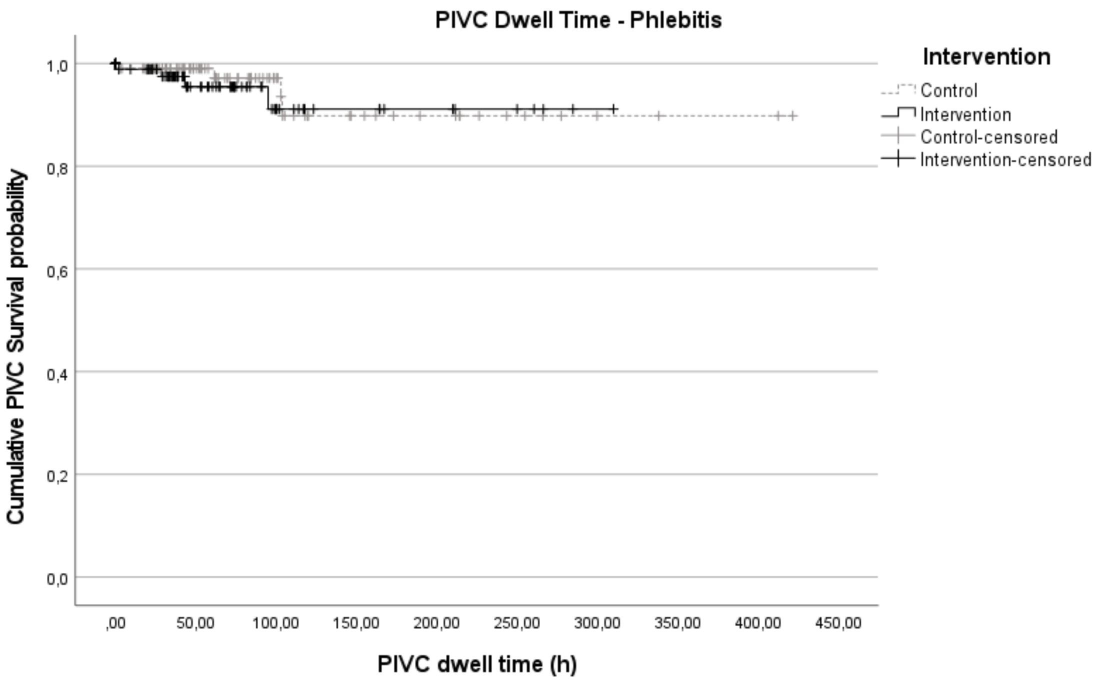

Fig 5. Kaplan-Meier diagram for all phlebitis, where Y axis indicate probability of PIVC survival (1 = 100% survival, 0 = 0% survival). X axis is dwell time in hours (h). Grey dotted line represent control, black whole line represent intervention, crosses on the line (censoring) represent a PIVC was removed before the longest PIVC dwell time observation. Log Rank test p=0.663.

S. Fig 6. Dislodgement

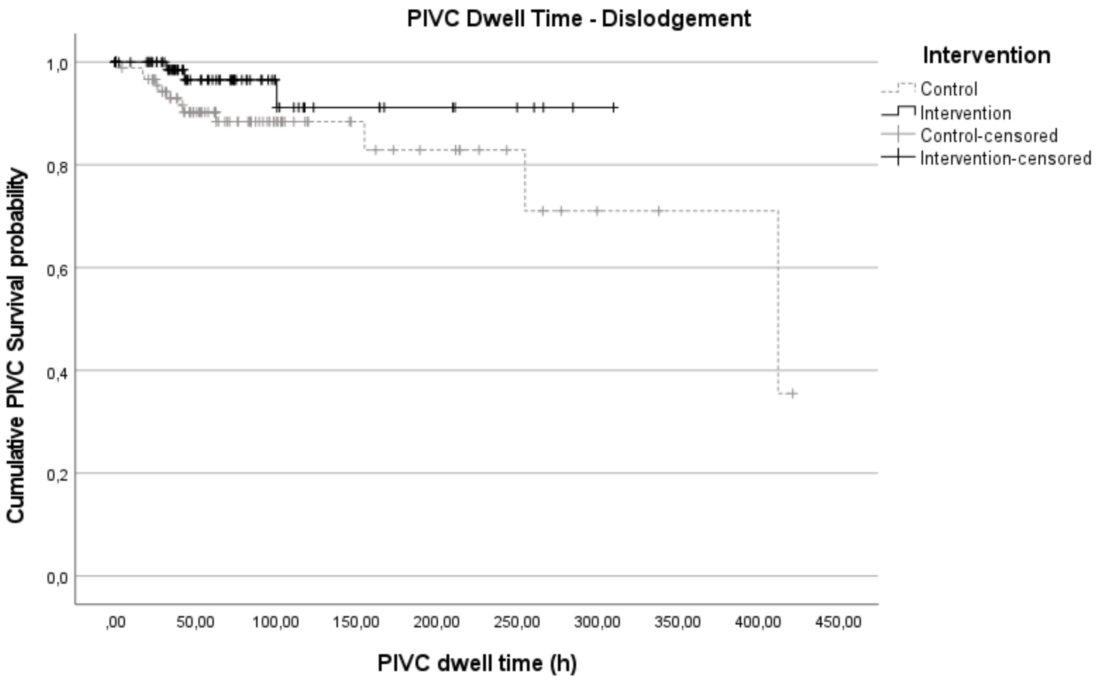

Fig 6. Kaplan-Meier diagram for all dislodgements, where Y axis indicate probability of PIVC survival (1 = 100% survival, 0 = 0% survival). X axis is dwell time in hours (h). Grey dotted line represent control, black whole line represent intervention, crosses on the line (censoring) represent a PIVC was removed before the longest PIVC dwell time observation. Log Rank test p=0.087.

S. Fig 7. Occlusion

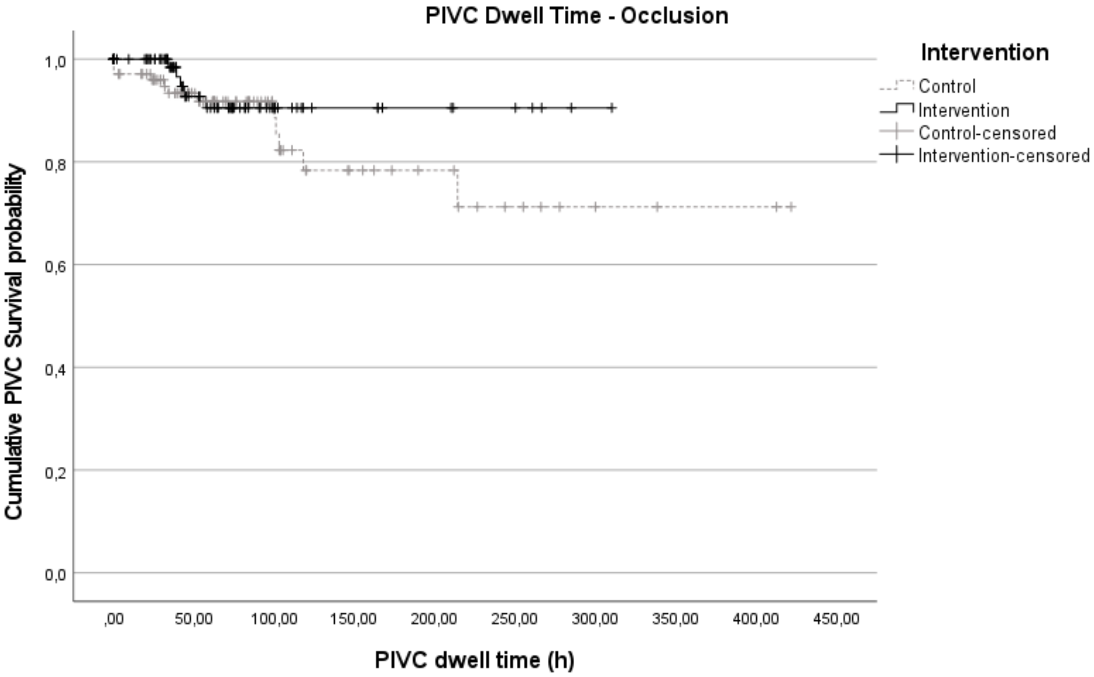

Fig 7. Kaplan-Meier diagram for all occlusion, where Y axis indicate probability of PIVC survival (1 = 100% survival, 0 = 0% survival). X axis is dwell time in hours (h). Grey dotted line represent control, black whole line represent intervention, crosses on the line (censoring) represent a PIVC was removed before the longest PIVC dwell time observation. Log Rank test p=0.283.

Fig 8. Leakage

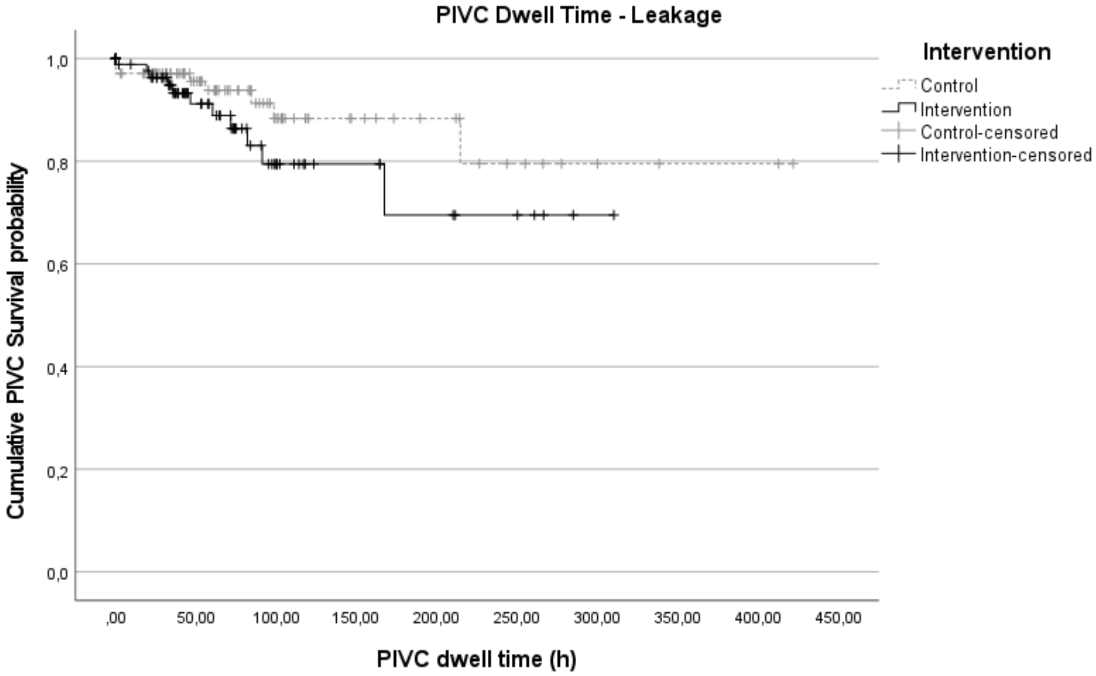

Fig 8. Kaplan-Meier diagram for all leakage, where Y axis indicate probability of PIVC survival (1 = 100% survival, 0 = 0% survival). X axis is dwell time in hours (h). Grey dotted line represent control, black whole line represent intervention, crosses on the line (censoring) represent a PIVC was removed before the longest PIVC dwell time observation. Log Rank test p=0.184.

S. Fig 9. Pain at injection

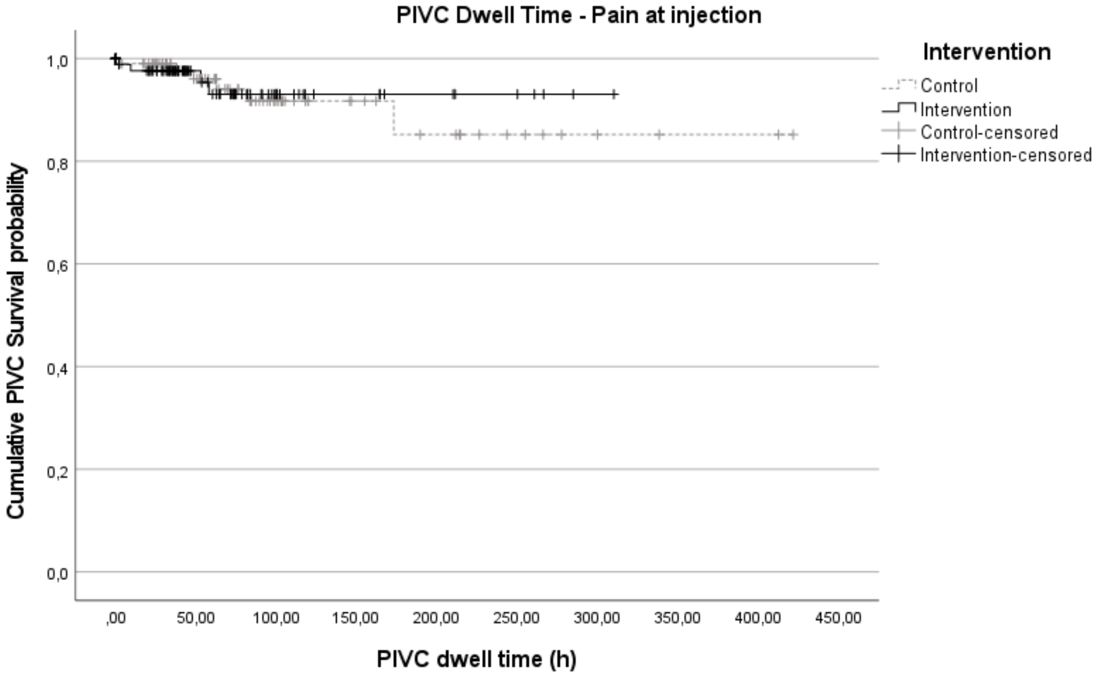

Fig 9. Kaplan-Meier diagram for all pain at injection site, where Y axis indicate probability of PIVC survival (1 = 100% survival, 0 = 0% survival). X axis is dwell time in hours (h). Grey dotted line represent control, black whole line represent intervention, crosses on the line (censoring) represent a PIVC was removed before the longest PIVC dwell time observation. Log Rank test p=0.857.

S. Table 6. Healthcare staff questionnaire

I would use the product again (Q1)	I would recommend the product to a colleague (Q2)	I would want my relatives to have access to the product in case of potential healthcare needs (Q3)
3.6	4.0	4.0

Average answer from questionnaire (0 = strongly disagree, 5 strongly agree).

S. Table 7. Material costs

Material costs (€)			
Material type	Average Cost	Min	Max
PIVC	1.31	0.68	4.77
Disinfectant	0.02	0.01	0.07
NaCl flush	0.38	0.22	1.56
Dressing	0.14	0.13	0.27
Extension	0.26	0.00	0.38
Total	2.11		

Average, minimum and maximum costs material usage of mechanical complications in Euro (€).

References:

1. EBM - Calculators. Accessed May 9, 2024. https://med.mercer.edu/libraries/mobile-ebm/calculators.htm
